# A State of the Art Review of Fillet Welded Joints

**DOI:** 10.3390/ma15248743

**Published:** 2022-12-07

**Authors:** Dinesh Lakshmanan Chandramohan, Krishanu Roy, Hafez Taheri, Michail Karpenko, Zhiyuan Fang, James B. P. Lim

**Affiliations:** 1 School of Engineering, The University of Waikato, Hamilton 3240, New Zealand; 2 Heavy Engineering Research Association (HERA), Auckland 2104, New Zealand; 3 Department of Civil and Environmental Engineering, The University of Auckland, Auckland 1010, New Zealand

**Keywords:** fillet welded joint, static strength, fatigue strength, thermal performance, hollow section

## Abstract

Fillet welded joints are commonly used in steel structures for various engineering applications such as buildings, bridges, railways, ships, and marine structures. Fillet welded joints are generally subjected to static and fatigue loading, resulting in failures of such welded joints. A number of experimental and numerical investigations on the strength and failure behaviour of fillet welded joints have been published. This paper presents a comprehensive review of research results on the static strength, fatigue life, and thermal performance of fillet welded joints. The review covers the various influential factors, such as loading direction, weld geometry, grades of steel, filler materials, welding process, weld penetration, strength mismatch of weld metal, and post-welded treatment. In total, 100 papers were critically reviewed, which were published from 1970 till date. The key findings and research developments on fillet welded joints are summarised. It was found that the transverse fillet welded joints have a higher static strength than the longitudinal fillet welded joints. Filler materials, post-welded treatment, and penetration of weld metal can offer significant strength enhancements in terms of their static and fatigue strength. Lastly, research gaps have been found in the existing body of knowledge, which will help guide future research.

## 1. Introduction

**Figure 1:**
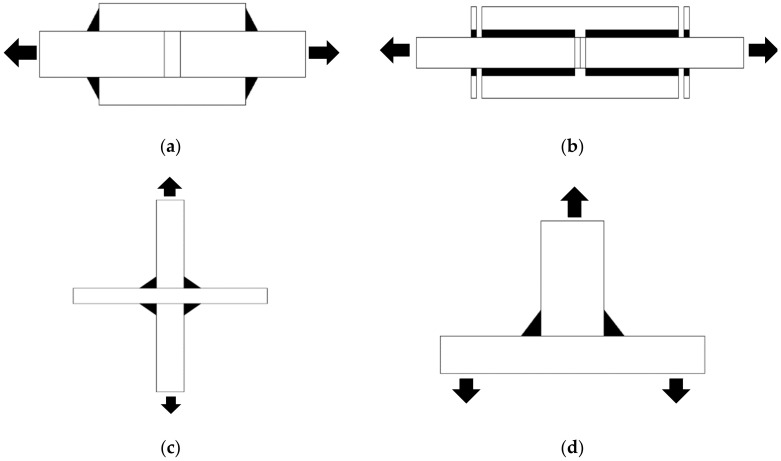
Schematic diagram of fillet welded joints. (**a**) Transverse fillet welded lap joint; (**b**) Longitudinal fillet welded lap joint; (**c**) Cruciform joint or X-joint; (**d**) T-Joint.

Fillet welded joints such as lap, cruciform and T-Joints are the most common connections in welded fabrication (see Figure 1). The widespread use of fillet welded joints in civil engineering applications, such as buildings, bridges, and marine structures, is attributable to their inherent advantages of fabrication simplicity, robot welding process, and cost-effectiveness [1,2]. The structural performance and failure mechanism of fillet-welded joints under various loading conditions (e.g., static loading, cyclic loading and fire loading) is complex and has received considerable academic attention. Extensive experimental and numerical studies have been conducted over the last three decades to investigate the behaviour of fillet-welded joints under various influencing factors. This study aims to gather the available research on the static strength, fatigue strength, and thermal performance of fillet-welded joints to identify the research gap and get a deeper understanding of their structural performance and failure mechanism.

The static design strength is of crucially great importance when applying fillet welded joints in the fabrication of structures. The static performance of fillet welded joints has been investigated using experimental tests, numerical simulations, and analytical methods [3]. There are numerous factors that affect the structural behaviour of fillet-welded joints under static loading: (a) loading direction (transverse, longitudinal and inclined); (b) weld size; (c) plate thickness; (d) weld penetration; (e) filler material; (f) steel grade; (g) strength mismatch of weld metal; and (h) contact gap size between two plates [4,5,6].

The fatigue behaviour (low-cycle fatigue and high-cycle fatigue) of fillet welded joints is important when designing such joints for structural applications involving cyclic loading conditions. The fatigue performance of fillet welded joints has been investigated through experimental tests, and numerical simulations [7]. There are two types of fillet welded joints in the available research: load-carrying joints and non-load-carrying joints. The fatigue strength and behaviour of both load-carrying joints and non-load-carrying joints are usually affected by: (a) thickness of steel plates; (b) weld size; (c) grade of steel; (d) weld penetration depth; (e) post-welded treatment; and (f) low and high cyclic loading [8,9].

The behaviour of fillet welded joints under fire loading is a topic that has recently attracted the attention of researchers. At elevated temperatures, the strength and behaviour of fillet welded joints become more complex due to changes in their material properties. Several studies [10,11,12,13,14,15,16] have explored the structural performance of the fillet welded joints at elevated temperatures (from 20 to 900 °C), using thermal experimental testing. In the experimental testing, various factors were considered, including temperatures, different loading rates, loading directions, and cooling methods to investigate the strength and behaviour at elevated temperatures [10,11].

Hence, this paper provides a critical review of the fillet welded joints, in order to identify the challenges and gain a better knowledge of their static structural performance, fatigue behaviour, and thermal performance. By further understanding the behaviour of fillet welded joints, researchers would be able to recognize the challenges of the fillet welded joints and conduct safe design solutions. In total, 90 papers based on fillet welded joints and research findings were critically reviewed. The existing design standards, experimental techniques, computational approaches, and research background were described for fillet welded joints under static, fatigue and fire loading. Various influencing factors on the structural behaviour of fillet welded joints are also summarised. Finally, the recommendations were mapped for future directions.

## 2. Static Design Strength of Fillet Welded Joints

The experimental and numerical studies were conducted to investigate the behaviour of fillet-welded joints in terms of design strength, failure angle, and fracture mechanisms. The following factors were investigated on the structural performance of fillet-welded joints: (a) loading direction; (b) weld geometry; (c) plate thickness; (d) weld metal; (e) filler material; (f) steel grade; (g) penetration depth; (h) strength mismatch of weld metal; (i) welding process; and (j) contact gap size between two plates. Table 1 presents the key information from the existing literature on fillet-welded joints under static loading.

### 2.1. Effect of Longitudinal and Transverse Loading Direction on Structural Behaviour and Static Strength

Many researchers investigated the structural behaviour and static strength of longitudinal and transverse fillet welded joints. Butler and Kulak [17] conducted experimental tests and numerical simulations on the transverse and longitudinal welded joints and found that the transverse weld is higher in strength by about 44% over the longitudinal weld [36]. Kamtekar [37] investigated the static strength of fillet welded joints including transverse, longitudinal and full round welded joints. It was found that the increase in the strength of the welded joints was about 50% as the angle between the weld axis and the loading direction increased from 0° to 90° degrees. Besides, some studies focused on the static strength and behaviour of the various grades of steel, the following research review summarises in terms of high-strength steel and stainless-steel fillet welded joints.

**Figure 2:**
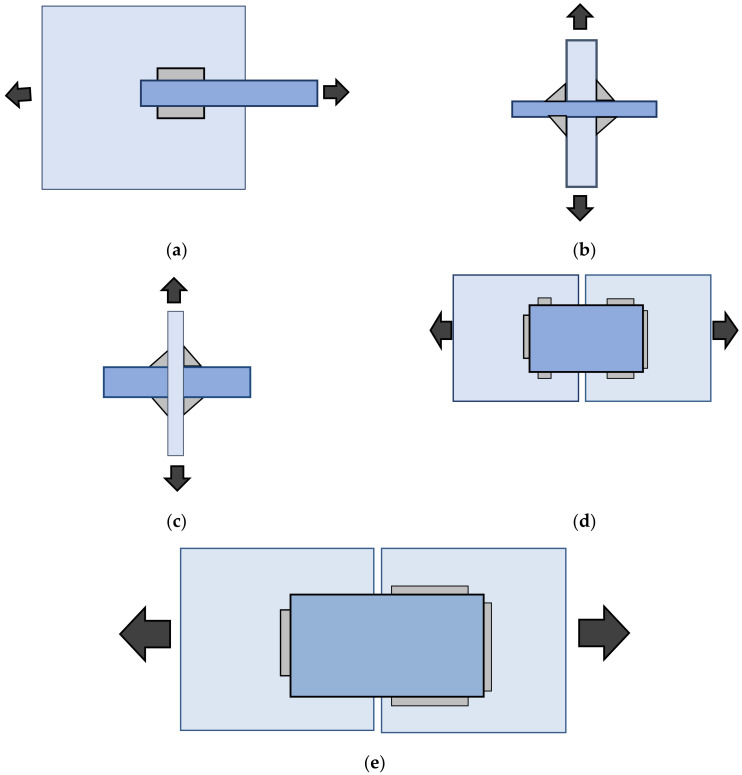
Different types of fillet welded joints. (**a**) Longitudinal loaded cruciform joint; (**b**) Load-carrying transverse cruciform joint; (**c**) Non-load carrying transverse cruciform joint; (**d**) Transverse and longitudinal load-carrying joint; (**e**) Transverse load-carrying lap joint.

In terms of high-strength steel, Collin and Johansson [28] studied the strength of fillet welded joints made of high-strength steel (S960) by conducting experimental tests on both longitudinal and transverse fillet welded joints under static loading. They also assessed the performance of existing design guidelines [38] by comparing the design strengths to test results, revealing that the current design rules [38] were underestimated in predicting the static strength of transverse fillet welded joints. Kuhlmann et al. [22] carried out experimental and numerical studies on high-strength steel (S690) fillet welded joints under static tensile loading and evaluated the feasibility of Eurocode 3 1993-1-8 [39] in predicting the static strength of such joints. Bjork et al. [23] investigated the ultimate load-carrying capacity of the high-strength steel S960 fillet welded joints using laboratory tests and numerical analysis. Various typical fillet welded joints were investigated, including longitudinal loaded cruciform joints, transverse loaded carrying lap joints, transverse and longitudinal load-carrying lap joints, load-carrying transverse cruciform joints, load-carrying transverse cruciform joints, and non-load-carrying transverse cruciform joints (see Figure 2) [23]. Similarly, Bjork et al. [1] also studied the moment capacity of the fillet welded joints. The four-point bending tests were performed on two types of welded joints (single weld and two parallel welds). It was found that the angle of failure was 20° to 30°, which is different from the theoretical assumption of an angle 45° [1].

In terms of stainless steel, Fortan et al. [26] conducted experimental tests to investigate the design strength of longitudinal and transverse fillet welded joints made of austenitic and duplex-grade stainless steel. The experimental results were compared with the current design guidelines of Eurocode 3 1993-1-4 [40], revealing that the correlation factor proposed in the design guidelines [40] was reliable to determine the static strength for austenitic specimens and slightly conservative for duplex-grade stainless steel. Similarly, the ultimate design strength and weld metal fracture mechanism of austenitic stainless steel fillet weld were studied by Lee et al. [27]. The test results were compared with the prediction of the ASCE specification [41] and they developed new equations to determine the design strength of the weld based on the test results. Yang et al. [30] conducted an experimental and numerical investigation on transverse and longitudinal duplex stainless steel fillet welded joints to study the design strength, deformation, and failure angle of the welded joints under static loading. The test results showed that the average fracture angle was 29° and 46° for transverse and longitudinal welded joints, respectively, while the ultimate design strength of transverse fillet welded joints was observed to be around 1.5 times greater than the longitudinal fillet welded joints. The experimental and FEA results were compared with the design strengths obtained from the current design guidelines of Eurocode 3 part 1993-1-4 [40], ASCE 8-02 [41] and CECS 410 [42] indicating that the design strengths were generally conservative. Based on the test and FEA results, they proposed a modified design formula to determine the static strength of duplex stainless steel fillet welded joints. Recently, Fortan et al. [32] carried out a total of twenty-four experimental studies on the static strength of transverse and longitudinal fillet welded joints with different grades of stainless steel and welding processes. They evaluated the applicability of the current design standards of Eurocode 3 1993-1-8 [39] and Eurocode 3 part 1993-1-4 [40] based on the available test data [27,30,32,43]. The reliability analysis showed that the current European design rules [39,40] provided conservative results. Therefore, a new correlation factor of 0.84 and 0.92 was proposed to determine the static strength of austenitic and duplex stainless steel fillet welded joints, respectively.

### 2.2. Effect of Inclined Loading Direction on Fillet Welded Joints

Several studies investigated the fillet welded joints under transverse and longitudinal loading conditions. It was found that the weld strength and behaviour depend on loading direction, therefore the effect of fillet welded joints under inclined loading direction is also an important concern in this field. Some studies [17,20,21,37,44] were performed to investigate the structural behaviour of transverse, longitudinal, and inclined fillet welded joints under static loading. The effect of different filler materials with the inclined angle of loading was studied [19,20]. Ng et al. [19] conducted detailed experimental tests on the transverse fillet welded joints under static loading, including lap and cruciform joints. Various influential parameters were considered: (a) filler metal, (b) weld size, (c) welding process (Shielded metal arc welding and flux cored arc welding) [19]. The test results were compared with the design guidelines of CSA S16 [45], AISC (1999) [46] and the feasibility of these design rules was investigated. In terms of various loading directions, inclined fillet welded joints showed higher static strength and a lower fracture angle than the longitudinal fillet welded joints, and vice versa for transverse fillet welded joints.

### 2.3. Effect of Weld Penetration and Strength Mismatch of Weld Metal

**Figure 3:**
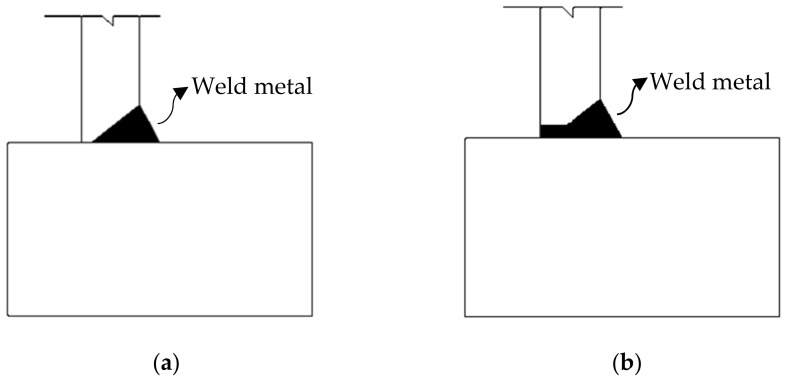
Penetration level of weld metal in fillet welded joints. (**a**) 75% penetration of weld metal; (**b**) 100% penetration of weld metal.

**Figure 4:**
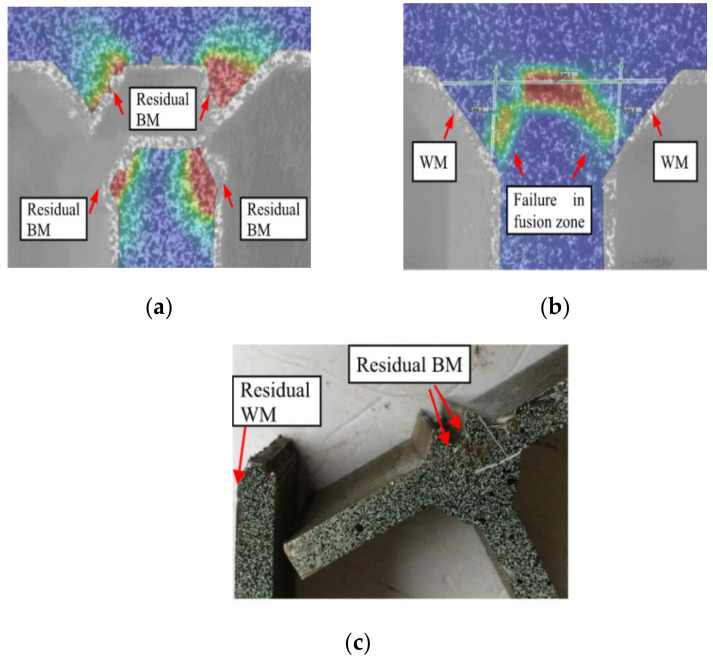
Failure mode of cruciform fillet welded joints obtained from DIC measurements and experimental (used with permission, Elsevier [29]). (**a**) Cruciform joint (CC1-5-1); (**b**) Cruciform joint (CC3-5-1); (**c**) Experimental.

The strength of fillet welded joints was significantly influenced by the penetration depth and strength mismatch of the weld metal. Khurshid et al. [5], and Barsoum and Khurshid [24] conducted experimental tests and numerical studies on cruciform fillet welded joints with full penetration (100%) and partial (50% and 75%) penetration of the weld metal (Figure 3). The results were compared with the design rules of Eurocode 3 1993-1-8 [39], AWS D1.1 [47] and BSK 07 [48] and indicated that the ultimate strength of the fillet welded joints was improved with increases in penetration percentage. Sun et al. [29,33] studied the mechanical behaviour of fillet welded joints including ultimate strength, failure mode, fracture angle, and weld ductility with the different parameters of weld size and mismatch ratio of the weld metal. These studies [29,33] showed that the increase in strength mismatch ratio increased the static strength of fillet welded joints. The average fracture angle of transverse lap joints, cruciform joints and longitudinal lap joints was angle of 20.1°, 18.5° and 51.5°, respectively. The fracture locations for the transverse and longitudinal lap welded joints were identified on the weld metal. In contrast, fracture location was observed in the fusion line area for cruciform joints (Figure 4), due to softening and other metallurgical effects. Moreover, the ductile fracture behaviour of transverse lap fillet welded joints was explored by Shi and Chen [25]. They conducted the experimental and numerical investigation on fillet welded joints built of high-strength steel plates with two different weld metals. The developed FE models were able to predict the accurate fracture angle of the fillet welded joints and they reported that the average fracture angles were from 16.5° to 19° for the transverse fillet welded joints. Recently, Ran et al. [35] carried out experimental and numerical studies on transverse fillet welded joints made of high-strength steel (SQ690) and studied their fracture behaviour. The results showed that the cracks developed at the root of the fillet weld and propagated to the weld metal in both cases of lap and cruciform fillet welded joints. The effect of weld penetration and strength mismatch ratio methods shows the strength enhancement in fillet welded joints under static loading.

### 2.4. Traction Stress Method

**Figure 5:**
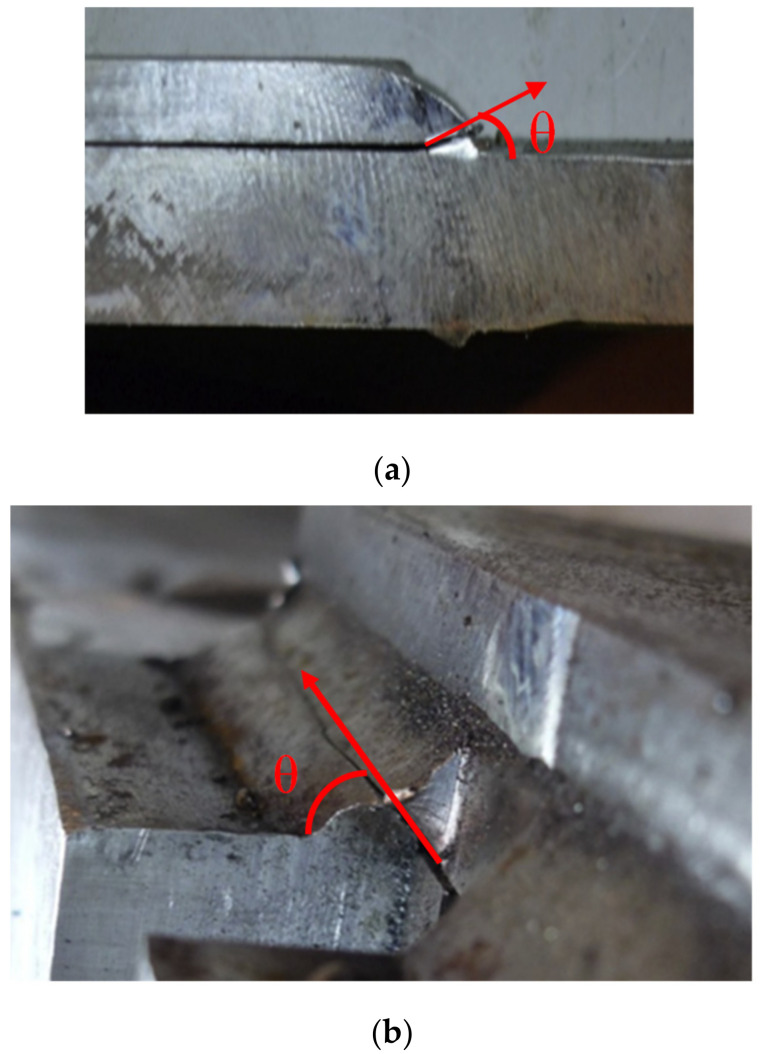
Failure angles for fillet welded lap joints (used with permission, Elsevier [3]). (**a**) Failure angle for transverse fillet welded joint; (**b**) Failure angle for longitudinal fillet welded joint.

The traction stress method is a stress analysis method capable of accurately extracting the stress parameters correlated to the static shear failures, which is needed in order to avoid discrepancies in failure path and shear strengths [3,49]. The traction stress method has been proven effective in determining the shear strength of fillet welded joints compared to the conventional shear stress method [3]. Nie and Dong [49] introduced a new traction shear stress definition and calculation procedure to evaluate the static shear strength of the fillet welded joints. Therefore, this study developed finite element (FE) models of transverse and longitudinal fillet welded joints and validated against the test results of McClellan [50] in terms of shear stress and failure angles. To gain theoretical insights, they presented the analytical solution for normal stress and member shear components. Both FE based and analytical solutions showed the exactly same for fillet welded joints under transverse shear conditions. In addition, Lu et al. [3] conducted experimental and numerical research on the tensile strength of welded joints using the traction stress method. This study examined the transverse and longitudinal fillet welded joints with various parameters including the base metal, filler metal, material strength, plate thickness, welding process, and weld size. The fillet weld size profile for the transverse and longitudinal fillet welded joints were designed according to the ASW B.4 [51]. It was found that the failure angles for transverse and longitudinal shear specimens are 22.5° and 45°, respectively (see Figure 5) [3]. In addition, they proposed design equations to determine the shear strength of the fillet welded joints.

### 2.5. Static Strength of Fillet Welded Joints Made of Thin Steel Plates

Under static loading, the strength and behaviour of fillet welded joints will be different for varying steel plate thicknesses. Some studies [4,18] were conducted to investigate the static behaviour of fillet welded joints made of thin steel plates. Teh and Hancock [18] investigated the strength of fillet welded joints built of thin steel sheets with thicknesses ranging from 1.5 mm to 3 mm. Four types of fillet welded joints were studied: single-lap transverse joints, single-lap longitudinal joints, double-lap transverse joints, and longitudinal joints. The test results were compared to the specifications for cold-formed steel structural elements such as AS/NZS 4600 [52] and AISI S100 [53], and it was found that the existing guidelines were unreliable to predict the strength. Similarly, Torabian et al. [4] conducted an experimental study on thin steel plates with double fillet welded lap joints under static tensile loading. The test results were compared with the design rules of AISI S100 [52] for the cold-formed steel structural members and the appropriate design equation was proposed to design the transverse fillet welded joints.

### 2.6. Weld Discontinuity on the Static Strength of Fillet Welded Joints

The weld discontinuities are unavoidable during the fabrication of welded joints [8]. The geometrical discontinuities such as weld root gaps, lack of fusion, the overlap between steel plates, and incomplete penetration are mostly associated with fillet welded joints. The weld discontinuities associated with fillet welded joints are difficult to detect and evaluate by visual inspection. The presence of geometric discontinuity decreases the load-carrying capacity of such fillet welded joint. Sachin and Vyavahare [6] conducted a finite element analysis to examine the effect of root gap discontinuity on the fillet welded T-joints that were eccentrically loaded and concluded a 50% reduction in the capacity of the fillet weld with the presence of a gap between the plates. Moreover, fillet welded joints with incomplete penetrations showed decreased static strength compared to full penetration under static loading [5,24]. It was observed that the presence of geometrical discontinuity on the fillet welded joints has an effect on its static strength.

### 2.7. Block Shear Strength of Fillet Welded Joints

**Figure 6:**
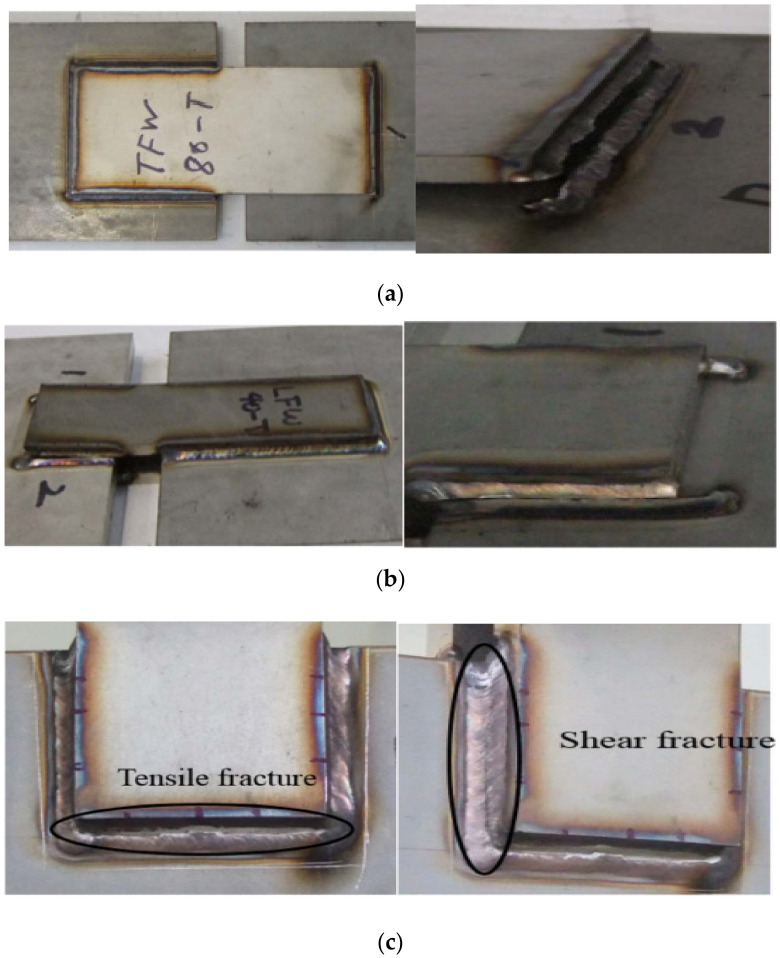
Block shear fracture for fillet welded joints [27]. (**a**) Transverse fillet weld (tensile fracture); (**b**) Longitudinal fillet weld (shear fracture); (**c**) Full fillet weld (block shear fracture: tensile fracture → shear fracture).

The block shear fracture of fillet welded joints is complicated and it occurs due to the combination of tensile and shear fracture mechanisms. In order to investigate the block shear strength and behaviour of fillet welded joints, Lee et al. [27,31] and Cho et al. [34] performed experimental and numerical research on austenitic and duplex stainless steel fillet welded joints, respectively. In both studies, full fillet welded joints were considered. The fracture mechanisms of tensile fracture, shear fracture and block shear fracture (see Figure 6) [27] were observed in the test results of transverse, longitudinal and full fillet weld joints, respectively. From the extensive investigation on block shear strength of austenitic fillet welded joints, Lee et al. [31] found that the design standards of Eurocode 3 [39], ANSI/AISC 360 [54], Oosterhof and Drivers [55] and Topakaya [56] showed a tendency to underestimate the block shear strength of fillet welded joints. Therefore, Lee et al. [31] proposed modified tensile and shear stress factors to estimate block shear strength for austenitic grade steel. Similarly, Cho et al. [34] compared the experimental results with the currently available design rules [31,34,53,56] and showed that the available design rules were unreliable to calculate the block shear strength for duplex stainless steel fillet welded joints. In their study, a new design equation was proposed to fix this gap.

### 2.8. Design Guidelines

The design guidelines of Eurocode 3 1993-1-8 [39], IIW (International Institute of Welding) [57], AWS D1.1 (American Welding Society) [47], ANSI/AISC 360 (American Institute of Steel Construction) [54], CSA S16 (Canadian Standard Association) [45], AISC (American Institute of Steel Construction) [46], and AS 4100 (Australian Standards) (1998) [58] provided the design equations to estimate the strength of fillet welded joints under static loading. Eurocode 3 part 1993-1-12 [59] extended the design rules for undermatching filler materials and ABS 96 [60] specified fillet weld design for marine structures. To determine the static design strength of thin steel sections, design rules of AS/NZS 4600 [52] and AISI S100 [53] were available for cold-formed carbon steel. The design specifications of Eurocode 3 part 1993-1-4 [40], ASCE 8-02 (American Society of Civil Engineers) [41], and CECS 410 (Chinese Standards) [42] specified guidelines for stainless steel. Moreover, AWS B4 [51] provided the weld size specification for longitudinal and transverse fillet welded joints under shear loading, and included the design equations for calculating the shear strength of such specimens. Indeed, the weld sizing criterion for fillet welded joints using the traction stress method was established based on the AWS B4 [51] formula with a modification to consider both stress concentration and the actual angle of failure.

### 2.9. Investigation Methods

**Figure 7:**
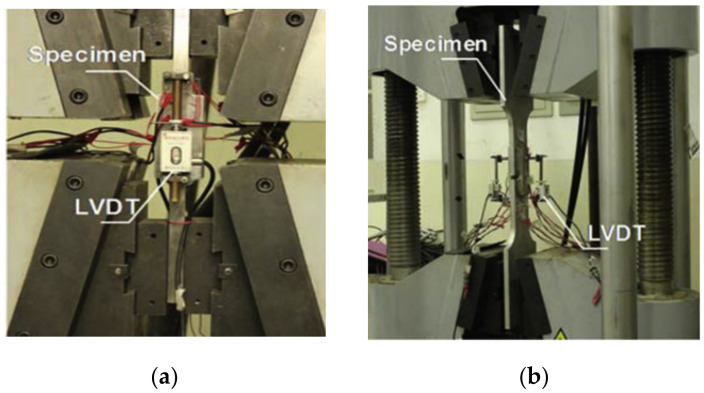

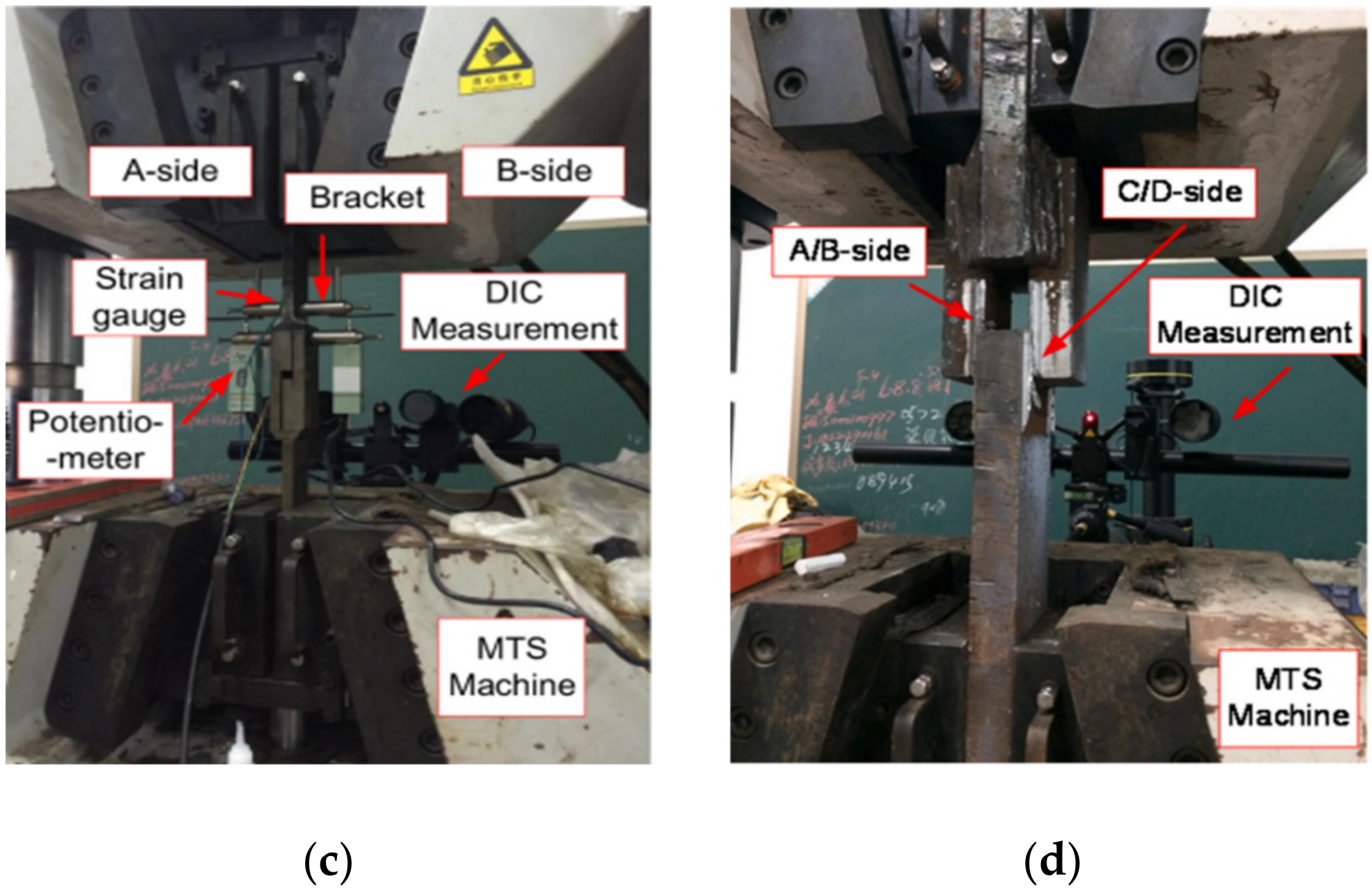
Experimental setup for fillet welded joints (used with permission, Elsevier [30,33]). (**a**) Transverse fillet welded joint; (**b**) Longitudinal fillet welded joint; (**c**) Transverse fillet welded joint; (**d**) Longitudinal fillet welded joint.

**Figure 8:**
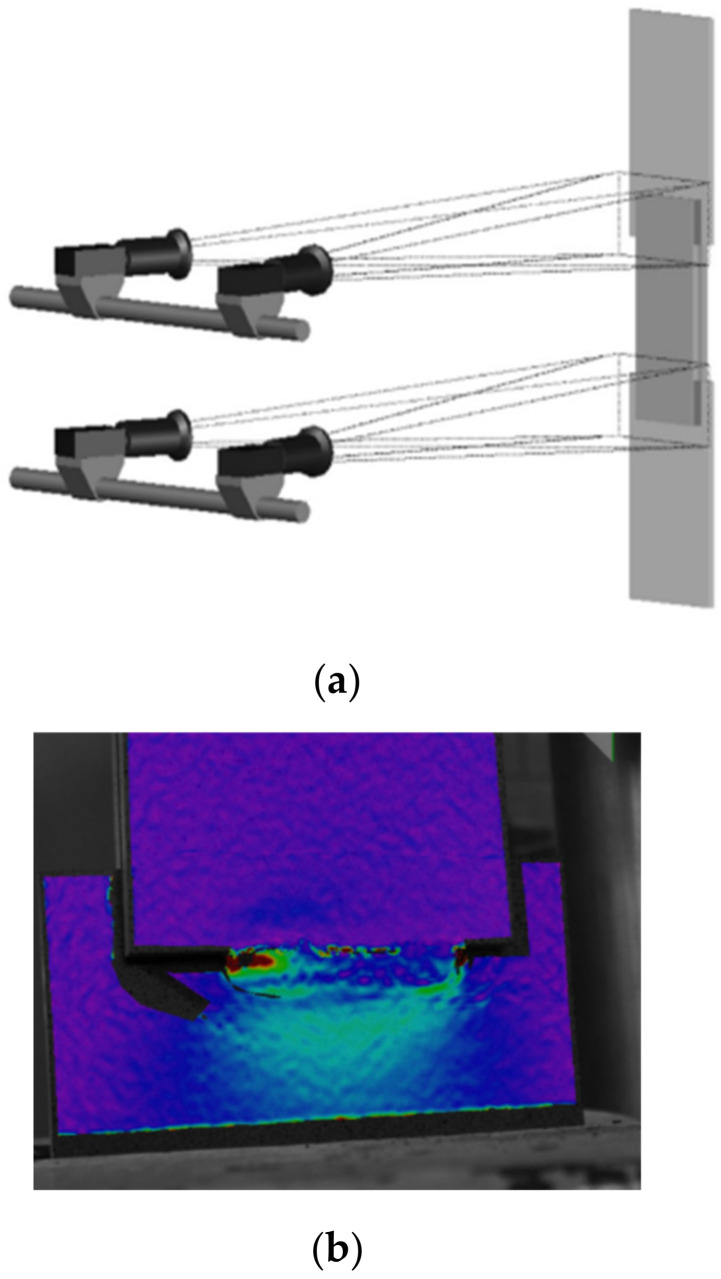
DIC setup and measurements for fillet welded joint (used with permission, Elsevier [29,32]). (**a**) DIC measurement set-up; (**b**) Measured strain pattern for transverse fillet weld.

To investigate the structural behaviour of the fillet welded joints under static loading, experimental and numerical methods were found in most of the studies. For an experimental investigation, a tensile test was conducted quasi-statically using a hydraulic testing machine to investigate the static strength and behaviour of fillet welded joints. In which, a concentric tension load was applied to the fillet welded joints until failure. Figure 7 illustrates the test setup for fillet welded joints under static loading. Electronic data acquisition was achieved by using a traditional transducer known as a linear variable differential transformer (LVDT) (see Figure 7a,b) and strain gauges (see Figure 7c), which were mounted on the test specimen to monitor the deformation and fracture behaviour of the fillet welded joints. However, in recent times, stereovision digital image correlation (DIC) has been used to record the strain pattern and fracture behaviour of weld metal accurately to measure the corresponding design strength (see Figure 7b,c and Figure 8) [26,29,32,33]. DIC measurements were suitable for recording the deformation behaviour of transverse [29] and longitudinal [33] fillet welded joints. It was found that the tensile experimental test method was more suitable to investigate the strength and behaviour of all the types of fillet welded joints including, cruciform and T-Joints under static loading.

In terms of numerical simulation, the numerical modelling of fillet welded joints under static loading is challenging. 3D and 2D modelling techniques were adopted to investigate the static strength of fillet welded joints using various software packages such as ABAQUS [3,24,25,35,53,55], ANSYS [6,30] and FEMAP [23]. Nie and Dong [49] and Lu et al. [3] conducted a numerical simulation to perform the traction stress-based analysis. They recommended a 3D solid element with a reduced integration model and a 2D model under plane strain conditions for longitudinal and transverse fillet welded joints, respectively. It was found that the 2D models have significant advantages over the 3D models, including, a significantly lower number of elements and lesser computational time, which help the 2D models to simulate accurate stress and strain distribution results [35].

## 3. Fatigue Behaviour and Strength of Fillet Welded Joints

The fatigue strength and behaviour of fillet welded joints are summarised in this section. The objective of most of the research studies available in the literature was to investigate the strength and behaviour of fillet welded joints under cyclic loading. Various effects on the fatigue strength of fillet welded joints were examined: (a) thickness of steel plates; (b) weld geometry; (c) grade of steel; (d) weld penetration depth; (e) post-weld treatments; and (f) low and high cyclic loading. The key information from the published paper on fillet welded joints under fatigue loading is presented in Table 2.

### 3.1. Effect of Steel Plate Thickness on the Fatigue Strength of Fillet Welded Joints

Kainuma and Kim [65] conducted experimental tests and developed analytical models for load-carrying cruciform fillet welded joints and extensively studied the effects of different main and cross steel plate thicknesses (6 mm to 25 mm). It was observed that higher fatigue strength is achieved when the main and cross plates are of the same thickness. Takena et al. [86] conducted an experimental investigation into the fatigue strength of transverse fillet welded joints. This research reported that the increase in the thickness of the main plate leads to a reduction in fatigue strength. In addition, Kainuma and Mori [8,67] investigated fillet welded cruciform joints with different plate thicknesses using experimental and finite element methods. It was found that the effect of plate thickness influences the location of crack initiation and crack size on the fillet welded joints. Vishnuvardhan et al. [72] evaluated the fatigue strength of fillet welded joints with two different plate thicknesses using tests and numerical simulation. In which, the FEA results were well correlated with the experimental data and the test results were compared with the S-N curve from the recommendation of Eurocode 3 part 1–9 [87]. It was found that the decrease in intermediate plate thickness reduces the fatigue strength of the fillet welded joints.

### 3.2. Effect of Weld Geometries on the Fatigue Strength of Fillet Welded Joints

Weld geometries will significantly affect the strength of fillet welded joints under cyclic loading. Some notable studies were conducted to investigate the effect of different weld geometries on the fatigue strength and behaviour of fillet welded joints. Caccese et al. [66] investigated the effect of different weld geometry profiles on fillet welded cruciform joints. The fatigue strength of the fillet welded joints with the improved geometrical profile was higher than the same weld size with the other profile. Nykanen et al. [88] investigated the effect of weld toe radius, flank angle and weld size on the fatigue strength of fillet welded cruciform joints under tensile stress. Similarly, Lee et al. [69] investigated the fatigue strength of fillet welded joints intentionally with varied weld geometry. These studies [69,88] revealed that increasing the weld toe radius, weld flank angle, and weld throat thickness resulted in improved fatigue resistance. In addition, Shiozaki et al. [9] recently investigated the effect of weld toe geometry on the fatigue life of ultra-high-strength steel fillet welded lap joints. This study included both experimental and numerical analysis.

### 3.3. Effect of the Weld Penetration on the Fatigue Strength of Fillet Welded Joints

The effect of weld penetration has a significant influence on the fatigue strength and behaviour of fillet welded joints. From the experimental and numerical investigation on load-carrying fillet welded joints, it was found that the fatigue strength of load-carrying fillet welded joints increased with increasing penetration depth [8,67,70]. In the cyclic bending load case, the fatigue strength of fillet welded joints showed improvement by increasing weld penetration depth with effective throat thickness [89]. For full and incomplete penetration on load-carrying and non-load carrying fillet welded cruciform joints under cyclic loading [85]. The comparison of experimental and FEA results was found in good agreement with the S-N curve derived from the current design guidelines of IIW (2013) [90] and DNV GL [91] and reported that the crack locations were different for load-carrying and non-load carrying fillet welded cruciform joints with penetration depth. In terms of incomplete weld penetration depth in fillet welded joints under low and high cyclic loading. It was observed that the effect of incomplete weld penetration depth was found to have a significant improvement in fatigue strength under low cyclic loading compared to high cyclic loading [7]. For failure mechanisms, the crack propagation path was different for fillet welded joints with incomplete penetration according to low and high cyclic loading conditions [7,92,93]. In terms of fatigue strength enhancement, the weld penetration method is recommended as suitable for load-carrying and non-load-carrying fillet welded joints.

### 3.4. Effect of the Post-Welded Treatment on the Fatigue Strength of Fillet Welded Joints

The post-welded treatment methods can improve the weld profile and residual stress level at the weld toe, which has a significant influence on fatigue strength. Several studies have found that investigate the effect of post-welded treatment methods on the fatigue behaviour of fillet welded joints. Skriko et al. [74] conducted experimental and numerical simulations to study the fatigue strength of ultra-high strength steel (UHSS) (S960) TIG-dressed cruciform joints. It was found that the IIW recommendation (2013) [90] for the TIG-dressed fillet welded joints was shown to be over conservative for UHSS in calculating fatigue strength. In terms of HFMI post-welded treatment, the fatigue strength of fillet welded joints with and without post-welded treatment was investigated in duplex stainless steel. The experimental results showed improvements in fatigue strength after the post-weld treatment by HFMI at low-stress ratios. However, no strength improvement was found at high-stress ratios [77]. Similarly, the post-welded treatment of weld toe machining showed a significant improvement in fatigue resistance compared to the as-welded condition in UHSS lap fillet welded joints [9]. Additionally, Skriko et al. [74], Mettanen et al. [81] and Ahola et al. [82,94] performed fatigue strength assessments on fillet welded joints with post-welded treatment in recent times. It was found that the experimental test data has a good correlation with the 4R approach results, whereas the conventional stress-based approaches are conservative in fatigue strength estimation. Lastly, these studies recommended that the 4R method enables fatigue strength prediction with good accuracy for both the TIG-dressed and HFMI-treated fillet welded joints.

### 3.5. Effect of Steel Grade on the Fatigue Strength of Fillet Welded Joints

There is a wide range of applications of fillet welded joints in the engineering field. Multiple research investigations studied the fatigue strength and behaviour of fillet welded joints made of different grades of steel, including normal-strength steel (mild steel), high-strength steel, and stainless steel [61,63,64,68,73,75,80,83,95]. Mecseri and Kovesdi [75,95] conducted fatigue tests on cruciform joints and flange gusset joints made of high-strength and normal-strength steel. The use of high-strength steel material in cruciform joints demonstrated greater fatigue strength than normal-strength steel. They also discovered that the application of HSS provides better fatigue strength for reduced weld size and plate thickness. For flange gusset fillet welded joints, both studies [75,95] concluded that there was no significant difference in fatigue strength and crack propagation rate between the high-strength and normal-strength steel. On the other hand, Peng et al. [83] observed semi-elliptical type fatigue crack behaviour in austenitic stainless steel, which differed from the fatigue crack behaviour of structural steel. It was found that austenitic stainless steel has higher fatigue strength than structural steel. The comparison of experimental results with the Eurocode 3 part 1–9 [87] and IIW (2016) [96], showed 52% higher fatigue strength for stainless steel than for structural steel. From these studies, it was observed that the high-strength steel and stainless-steel fillet welded joints experience higher fatigue life compared to the normal-strength steel.

### 3.6. Effect of Low and High Cyclic Loading on Fatigue Analysis

In fatigue analysis, the fillet welded joints experience different fatigue strengths and failure mechanisms under low-cycle and high-cycle loading conditions. Therefore, some studies investigated the effect of low-fatigue and high-fatigue behaviour on fillet welded joints. Under low-cycle loading conditions, the crack initiation can be easily observed around the weld root at the early stage, and thus it can be concluded that the low-cycle fatigue strength was affected mainly by crack propagation [2,78]. Additionally, the fillet welded joints with incomplete penetration and strength mismatch, the fatigue behaviour of such joints was significantly affected under low-cyclic loading. However, in high-cycle loading, such effects can be negligible. Meanwhile, the crack propagation path was different, compared to the low-cycle loading case [7,92,93,97]. According to the fatigue strength assessment, the recently improved effective notch stress method shows good agreement with the test results in terms of predicting the fatigue strength and failure location for low-cycle and high-cycle loading [98].

### 3.7. Weld Dicontinuty on the Fatigue Strength of Fillet Welded Joints

Fatigue cracks can be initiated leading to fatigue failure in a fillet-welded joint with root discontinuity and penetration defects [85]. The geometrical weld discontinuity on the fillet welded joints intensifies the stress concentration effect and hence decreases the fatigue strength of such fillet welded joints. Miki et al. [99] investigated the fatigue strength of a fillet welded joint with root gap defects of various sizes and leg lengths. It was found that the root gap with penetration of weld metal increases the fatigue strength and suggested that the root gap of up to 3 mm will not lower the fatigue strength. In terms of penetration defects, the fatigue strength of fillet welded joints with incomplete penetration was decreased [85]. As a result, fillet welded joints are frequently subjected to careful inspection during fabrication in order to avoid weld discontinuities in the welded joint.

### 3.8. Effect of Sub-Zero Temperatures on the Fatigue Strength of Fillet Welded Joints

At sub-zero temperatures (below 0 °C), the material fracture mechanism can be changed and the fatigue strength of fillet welded joints will be different compared to the room-temperature (20 °C). Very limited research available in the literature investigated the effect of sub-zero temperatures on the fillet welded joints. The significantly higher fatigue strength was observed at sub-zero temperatures for fillet welded joints, and this is mainly due to the transition of ductile to brittle fracture mechanisms [79]. As for the detailed effects of different sub-zero temperatures, Braun et al. [79] investigated such effects using experimental tests. They found that the fatigue strength of cruciform fillet welded joints at temperatures of −20 °C and −50 °C increased by approximately 8% and 20%, respectively, compared to room temperature. The fatigue strength assessment based on available experimental data for fillet welded joints revealed that local stress-based methods were highly conservative in predicting fatigue strength and failure behaviour at sub-zero temperatures [100]. However, in order to give the right direction for the design work, the design guidelines for such joints at sub-zero temperatures should be more specific. Therefore, more experimental and numerical efforts on fillet welded joints at sub-zero temperatures are required.

### 3.9. Design Guidelines

The fatigue resistance for welded structures is based on the nominal design stress, utilizing the S-N curve. The design guidelines of EC3 part 1–9 [87], EC3 part 1993–1-4 [40], IIW [96] and BS 7608 [101] and provided the S-N curve to evaluate the fatigue life of fillet welded joints. EC3 part 1–9 [87] was the most commonly used to evaluate the fatigue strength of steel structures. These guidelines were widely applicable to various steel grades including carbon steel, stainless steel, and weathering steel. Moreover, IIW [96] design rules were considered as a universal curve for all types of welded structures.

### 3.10. Investigation Methods

Experimental and numerical methods were used to investigate the fatigue strength and behaviour of fillet welded joints. In the experimental testing, the cyclic load was applied to fillet welded joints in terms of tensile loading [7,8,62,65,73,75,76,77,81,83,85,95,100] and flexural loading [9,63,64,68,78,82,102] methods. In both cases, the cyclic load was applied to the fillet welded joints using a hydraulic actuator and a force transducer was used to determine the minimum and maximum fluctuation load. To perform the fatigue analysis, the fillet welded joints were loaded with varying stress ratios (R) and amplitudes. The load-control technique was used to conduct a high cyclic fatigue test on fillet welded joints, and a low cyclic fatigue test was performed by the displacement-control method [2,7,93]. Furthermore, various local stress-based approaches were employed to validate the experimental results in terms of strength and failure behaviour, such as the nominal stress method (NSM) [62,82,102], hot spot stress method (HSSM) [66,73,80,81,84], effective notch stress approach (ENS) [73,80,95,97,102,103] and 4R method [77,81,82,94]. Based on recent studies, the 4R technique is highly recommended to evaluate the fatigue strength of fillet welded joints with the effect of post-welded treatments.

In the numerical investigation, the FE model of fillet welded joints was developed using the software packages, including ABAQUS [2,63,74,76,80,83,84,93,103], ANSYS [66,70,78,81,95] and COSMOS [67,68]. The numerical modelling of fillet welded joints was challenging. There are two distinct types of numerical simulation methods were adopted, such as 2D modelling and 3D modelling technique. In the fatigue analysis, elastoplastic plain-strain 2D modelling approach [8,9,63,65,66,67,68,73,74,81,83,93] and 3D modelling techniques [2,71,76,80,84,85,95] were adopted to simulate the fillet welded joints under cyclic load case. The majority of the studies employed the 2D models to investigate the fatigue strength because of their advantages, such as a lower number of elements and less computational time than the 3D models.

## 4. Thermal Performance of Fillet Welded Joints

Some notable studies available in the literature investigated the strength and behaviour of fillet welded joints under elevated and post-elevated temperature conditions, which are summarised in this section. These studies accommodate the various influencing parameters such as loading direction, loading rates, temperatures and cooling methods on the strength of fillet welded joints. Table 3 presents the key information from the available studies on the fillet welded joint at elevated and post-elevated temperatures.

### 4.1. Thermal Behaviour of Fillet Welded Joints

At elevated temperatures, the thermal performance of the fillet welded joints is governed by loading rates, loading direction (transverse, longitudinal and inclined), and temperatures. The strength and stiffness of the fillet welded joints are decreased when the temperatures are increased [12], this indicates the temperatures have a significant influence on the strength reduction. In terms of loading rates, Ghor et al. [10,13] investigated the rate-dependent behaviour of fillet welded joints with different loading rates (fast and slow loading rates) at elevated temperatures. It was found that the loading rates have a significant effect on the strength of the fillet welded joints. Compared to the fast loading rate, the slow loading rate resulted in an 11–29% decrease in strength at a temperature increase from 475 °C to 700° C [13]. At elevated temperatures, the transverse fillet welded joints (90°) showed higher strength than the longitudinal (0°) and inclined fillet welded joints (45°) [10].

In post-elevated temperatures, the research findings are different from the aforementioned research. Usually, researchers pay much attention to the strength of fillet welded joints with different loading direction and cooling methods. Similar to the elevated temperature conditions, the fillet welded joints experienced a decrease in strength and stiffness as the temperature increased at the post-elevated condition [12]. In terms of different loading direction, the transverse, longitudinal and inclined fillet welded joints experienced a reduction in strength at higher temperatures. However, the reduction in the strength of fillet welded joints is around 25%, 22%, and 27% for the transverse, longitudinal and inclined loading direction at 900 °C, respectively. It showed that the effect of loading direction is negligible at post-elevated temperature conditions [14]. In terms of different cooling methods, the water-cooling fillet welded joints resulted in higher strength, stiffness and ductility than the natural cooling fillet welded joints at higher temperatures [11]. However, the structural behaviour of fillet welded joints becomes more complex, as compared to the ambient temperature research, because of changes in the material properties at fire exposure [15].

### 4.2. Experimental Methods

There are two types of experimental methods to investigate the thermal performance of fillet welded joints at elevated temperatures, such as transient-state and steady-state methods. In the transient state method, the fillet welded joints were initially loaded to a predefined tensile load and the specimens were subjected to a fire condition in the furnace while a constant load was applied until failure occurred [15]. The effect of temperatures and loading direction was investigated using this transient state method. On the other hand, the steady-state method is used to evaluate the loading rate-dependent behaviour of fillet welded joints at elevated temperatures [10,13]. In the steady-state, the fillet welded joints were heated in the furnace to the required temperature, after that, the displacement-controlled load was applied to the specimens at the rates of 1.5 mm/min (fast) and 0.1 mm/min (slow). According to the studies [10,13], it was found that this steady-state method is more suitable to conduct rate-dependent behaviour analysis on fillet welded joints at elevated temperatures. To investigate the thermal behaviour of fillet welded joints at post-elevated temperatures, the experimental test approach is similar to the ambient temperature except for the heating phase. In this test method, the fillet welded joints were heated to the appropriate temperature in the furnace and held constant for some time to ensure that the test specimens were uniformly heated. After removing the specimens from the furnace, they were allowed to cool and loaded (in tension) until failure, using a force-control loading technique [11,16]. The structural behaviour of different cooling methods was studied using this experimental approach.

### 4.3. Design Guidelines

AISC [18], Chinese specification (CECS) [104] and Eurocode 3 part 1–2 [105] provided the design guidance and retention factor for fillet welded joints at elevated temperatures. However, there is no specification or retention factor available for fillet welded joints under post-fire exposure conditions. In addition, the design equation of ANSI/AISC 360 [18] was modified to account for the loading rates and loading direction to determine the nominal strength of the fillet welded joints at elevated temperatures [13]. Moreover, AWS A5.1 [106] design specification provides the guidelines for the welding process.

## 5. Fillet Welded Joints on Hollow Sections

**Figure 9:**
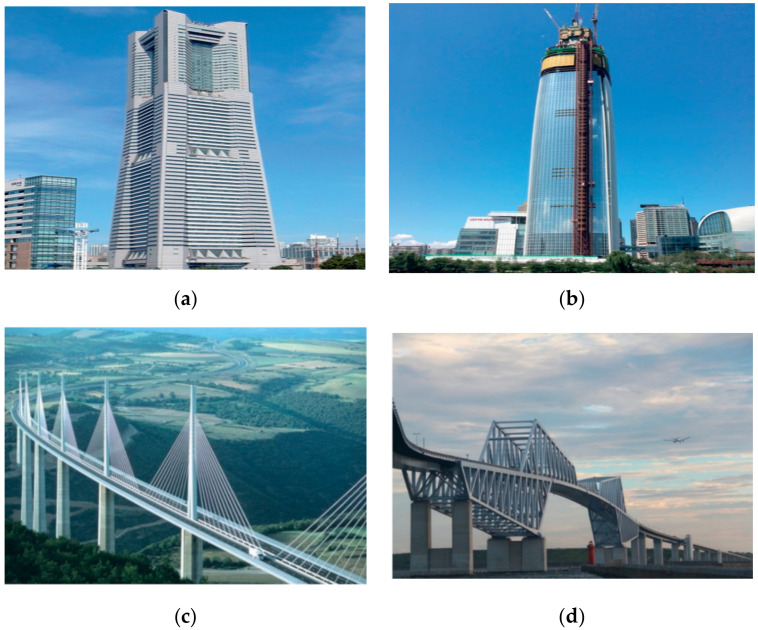
Structural applications of hollow sections (used with permission, Elsevier [107]). (**a**) Landmark tower, Yokohama; (**b**) Lotte world tower, Seoul; (**c**) Millau bridge, Millau-Creissels; (**d**) Tokyo gate bridge, Tokyo.

**Figure 10:**
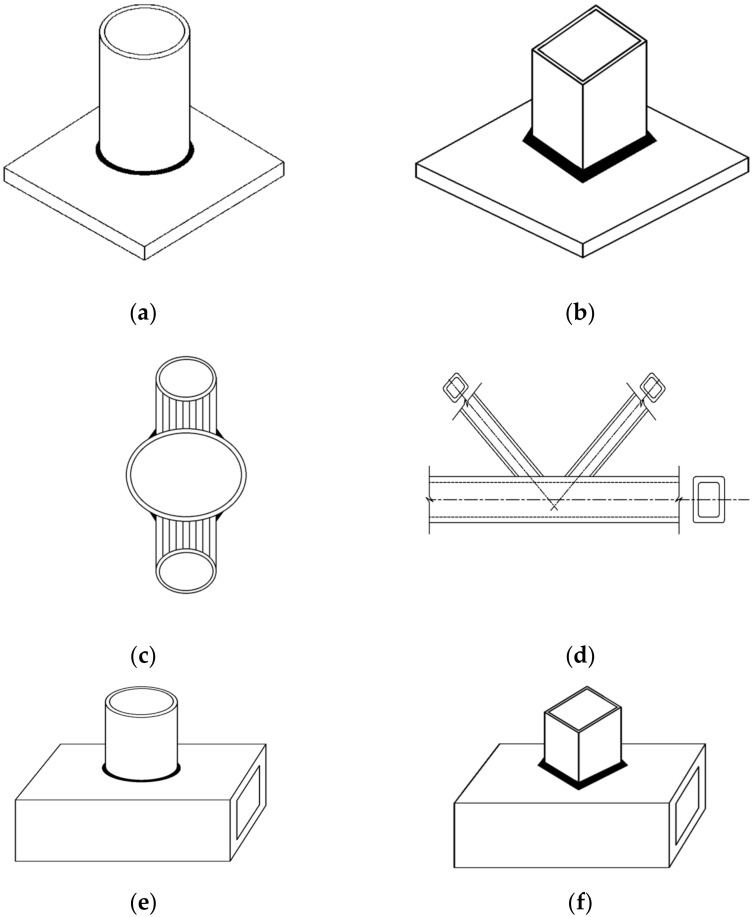
Various fillet welded joint connections on hollow sections. (**a**) CHS-to-plate connection; (**b**) SHS-to-plate connection; (**c**) CHS-to-CHS X-connection; (**d**) RHS-to-RHS K-connection; (**e**) CHS-to-SHS T-connection; (**f**) SHS-to-SHS T-connection.

Hollow sections were widely employed as structural members in onshore and offshore constructions such as high-rise buildings, long-span structures, offshore platforms, and bridges (see Figure 9) [107]. The hollow sections are majorly available in the form of Rectangular Hollow Sections (RHS), Circular Hollow Sections (CHS) and Square Hollow sections (SHS). Several researchers have studied the static strength [108,109,110,111,112,113,114,115] and fatigue strength [116,117,118,119,120,121] of fillet welded joints on hollow sections, including different joint geometries such as CHS-to-plate, RHS-to-plate, CHS-to-CHS X-connection, RHS-to-RHS K-connection, CHS-to-SHS T-connection, and SHS-to-SHS T-connection (see in Figure 10) [110,112,116,119,121]. Table 4 and Table 5 provides the important information from each of the relevant studies on the hollow sections under static and fatigue loading, respectively.

### 5.1. Experimental Methods

The direct tensile test and cyclic in-plane bending methods are adopted to investigate the static and fatigue strength of the fillet welded joints on hollow sections, respectively. In terms of static strength tests [108,110,111,113,114,115], the tensile load was applied quasi-statically using a universal testing machine to the specimen until the failure of the fillet welded joint with the displacement control method. The strain gauge and LVDTs were used in the test set-up to measure the stress distribution and load–displacement relationship of the fillet welded joints. On the other hand, to investigate the fatigue strength, the test specimens were subjected to in-plane bending cyclic loading [116,117,118,119,120,121]. Fatigue tests were conducted under load control methods with different loading frequencies (0035 to 1 HZ) and stress ratios (0.5 to 0.1).

### 5.2. Numerical Methods

A finite element analysis (FEA) approach was adopted to evaluate the strength and behaviour of fillet welded joints on hollow sections under static loading [109,110,112,115] and fatigue loading [121], using software packages such as ABAQUS [112,115] and Ansys [109,110,121]. To investigate the hollow sections, non-linear 3D and 2D FE models were adopted. It was found that most of the research preferred the 3D modelling technique [109,110,112,121] than the 2D models [115] in recent years. In order to reduce the computational time, half or quarter 3D models of actual geometries were adopted, which can be able to perform the analysis with minimal time and give results with good accuracy [109,110,121].

### 5.3. Static Strength of Fillet Welded Joints on Hollow Sections

**Figure 11:**
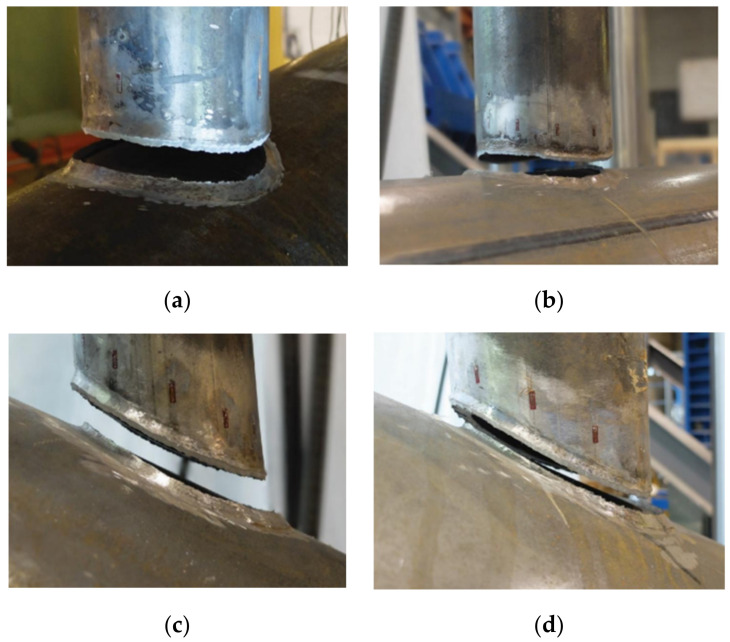
Typical failure modes of fillet welded joints on CHS-to-CHS connections (used with permission, Elsevier [108]). (**a**) Test 102–273-90a (θ = 90°); (**b**) Test 127-406-90a (θ = 90°); (**c**) Test 102-406-60a (θ = 60°); (**d**) Test 102-406-60a (θ = 60°).

The static strength and behaviour of fillet welded joints on hollow sections were investigated with different geometry sections including hollow section to hollow section (CHS-to-CHS and RHS-to-RHS) and hollow section to plate connections (CHS-to-plate and RHS-to-plate). In terms of hollow section to hollow section connections, Tousignant and Packer [108,109,110] conducted an experimental and numerical study on the fillet welded CHS-to-CHS X connections with various parameters. AWS [44] provided weld effective length design guidance for CHS-to-CHS-X connections. It was found that the branch-to-chord diameter ratios, branch-to-thickness diameter ratios and chord wall slenderness have a significant effect. However, the branch inclination angle has a negligible effect on the weld effective length. Figure 11 presents the typical failure mode of CHS-to-CHS X connections, the fillet welded joints failed by weld rupture along the plane of the weld metal [108,111]. The current design standards of AWS [44], AISC [50] and CSA [122] were evaluated by comparing the design strengths to FEA results, which showed that they are conservative [109]. Therefore, Tousignant and Packer [109] recommended an alternative method based on weld effective length to estimate the strength of fillet welded joints on the CHS-to-CHS X connection. Recently, Xin et al. [112] conducted an extensive numerical investigation on K-joints of RHS sections with various parameters. From the numerical FEA results, it was found that the secondary bending stresses increased as the strength of the material increased, the brace-to-chord width ratio increased, and the gap size of joints decreased.

For hollow section to plate section connections, Jiao and Zhao [113] and Ling et al. [114] investigated the static strength of fillet welded joints on CHS-to-plate made of very high strength (VHS) steel. A significant strength reduction was observed in the heat-affected zone (HAZ) of both transverse welded joints [113] and longitudinal fillet welded joints [114]. In addition, Zhao et al. [115] investigated the RHS-to-plate fillet welded joints under static loading. The experimental results were compared with the design strengths of AISC (1993) [123], AS 4100 (1990) [124], and CSA M89 [125] for weld metal strength and the design strengths of AISI (1996) [126], AS/NZS 4600 [127], CSA M94 [128] for base metal strength, which showed that they were not reliable. Therefore, new design equations were proposed to determine the static strength of RHS-to-plate fillet welded joints.

### 5.4. Static Strength of Fillet Welded Hollow Joints with Reinforcement

When the fillet welded hollow joints are subjected to static loading, the welded joint may fail due to the interaction of the brace and chord members. Several reinforcement techniques have been introduced, such as the collar plate, doubler plate, stiffener ring and fiber reinforced polymer (FRP) to improve the static capacity of the hollow joints. Nassiraei et al. [129,130,131,132] conducted the experimental and numerical investigation on the effect of collar plate reinforcement on the static strength of CHS fillet welded joints. In terms of compression, tensile, and in-plane bending, the reinforced hollow fillet welded joints showed significant strength gains over the unreinforced joints. In addition, Nassiraei et al. [133,134,135] and Choo et al. [136] studied the structural behaviour of CHS fillet welded X and T/Y-joints with doubler plate reinforcements. It was observed that the doubler plate reinforcement provides the strength enhancement (more than 200%) to both CHS-X and T/Y-joint. Moreover, the external stiffener ring reinforcement technique also provides a significant strength improvement to the CHS fillet welded X and T-joints under tensile [137,138] and compression loading [139,140] compared to unstiffened joints. Recently, the structural behaviour of FRP reinforced [141,142] CHS fillet welded joints was investigated. It was found that the use of FRP significantly increases the static load bearing capacity. All available reinforcement techniques are suitable for fillet welded hollow joints and significantly increase the ultimate strength of such joints.

### 5.5. Fatigue Strength of Fillet Welded Joints on Hollow Sections

In order to investigate the fatigue strength and behaviour of fillet welded joints on hollow sections, different geometrical connections were considered including hollow section to plate connections (SHS-to-plate and CHS-to-plate) [116,117,118,119] and hollow section to hollow section. The effects of in-line galvanizing, steel grade and stress ratio were investigated on the fatigue behaviour of SHS-to-plate fillet welded joints [116]. It was found that the steel grade and in-line galvanizing have negligible effects on fatigue strength. However, the effect of the stress ratio has a significant influence on fatigue strength [116]. From the experimental results [116,117,118,119], it was found that the fatigue failure mode of brace-tension side failure is observed in both SHS-to-plate and CHS-to-plate fillet welded joints. Besides, the fatigue strength of CHS-to-plate joints was shown to be higher than that of SHS-to-plate joints [119]. In terms of hollow section to hollow section connection, the fatigue cracks were found in either the chord or brace member or in both the chord and brace member under cyclic loading [120,121].

### 5.6. Fatigue Strength of Fillet Welded Joints with Reinforcements

To improve the fatigue strength of fillet welded hollow sections, few studies [143,144,145] investigated the FRP strengthening technique on the hollow joints. Hosseini et al. [143] conducted an experimental and numerical investigation on CHS T-joints with FRP under cyclic loading. It was observed that the stress concentration factor (SCF) for FRP reinforced joints is reduced by about 27% compared to unreinforced CHS T-joints. Similarly, the SCF for CHS fillet welded hollow joints is decreased by about 34% and 23% for compressive [144] and bending [145] fatigue loading, respectively. They proposed new reliable design equations to predict the SCFs in the CHS hollow joints with FRP reinforcement under fatigue loading [144,145]. The FRP reinforcement technique can be recommended to the fillet welded hollow joints in terms of fatigue strength enhancements.

### 5.7. Thermal Behaviour of Fillet Welded Joints on Hollow Sections

At elevated temperatures, Yu et al. [146] and Chen et al. [147] conducted an experimental study on the fillet welded circular hollow sections. It was found that the local buckling of chord wall for CHS T-joints at elevated temperatures showed similar failure mode obtained at ambient temperatures [146]. In order to improve the fire resistance, the effect of collar plate reinforcement on the static strength of SHS [148] and CHS [149,150,151,152] fillet welded joints at elevated temperatures were investigated. The fire resistance and ultimate strength of hollow T-joints have significantly increased with the reinforcement of collar plates and doubler plates. Moreover, the doubler plate offers higher initial stiffness than the collar plate at elevated temperatures. Pandey and Young [153] and Xu et al. [154] studied the strength and behaviour of fillet welded hollow joints with and without reinforcement plate at post-elevated temperatures and it was found that the doubler plate reinforcement has significant effect on fire resistance of fillet welded SHS T-joints.

## 6. Key Findings

In terms of fillet welded joints, the key findings and research improvements of the publications are briefly summarised below.

### 6.1. Static Loading

The static strength of fillet-welded joints is primarily investigated using experimental tests, numerical simulation and analytical methods. In terms of experimental tests, the fillet welded joints failed at weld metal, as confirmed by multiple experimental studies. For numerical simulation, the researchers have investigated the static strength and failure behaviour of fillet welded joints using a non-linear 3D and 2D model approach, however, it was observed that the FE modelling was not as accurate as expected. Nie and Dong [48] introduced a traction stress method to investigate the static strength of fillet welded joints. It was found that the traction stress method can accurately predict the strength and failure plane of fillet welded joints, as confirmed by experimental results. Besides, the effects of load direction, weld geometry, steel grade, filler material, weld penetration depth, strength mismatch of weld metal and welding process were also investigated in multiple research studies. In fillet-welded joints, the factors including load direction, filler material, strength mismatch of weld metal and weld penetration depth were significant. Meanwhile, duplex stainless steel possesses a slightly higher static strength than austenitic stainless steel.

### 6.2. Fatigue Loading

Fatigue strength is the main concern of the current study in the field of fillet welded joints. The fatigue strength and behaviour were investigated experimentally and numerically. Regarding experimental tests, the fillet welded joints were investigated under cyclic loading with different stress ratios and frequency rates. Various influencing parameters were considered in the fatigue test design, including the grade of steel, thickness of steel plate, weld geometry, weld penetration depth, low and high cycle loading, and post-weld treatment. It was found that weld penetration depth and post-weld treatment increased fatigue strength significantly. The fatigue life of fillet welded joints was mainly affected by the main plate thickness and grade of steel. High-strength steel and stainless steel experience better fatigue life than mild steel. Furthermore, at sub-zero temperatures, the fatigue strength of fillet-welded joints was higher than at ambient temperature. As for numerical simulation. both 3D and 2D simulation techniques were employed by researchers to investigate fatigue strength and behaviour, based on multiple published studies, it can be concluded that fillet welded joints can be simulated directly using 2D modelling technique accurately in terms of stress intensity factor and failure mechanism.

### 6.3. Thermal Performance

The thermal performance of fillet welded joints at elevated temperatures is investigated mainly by experimental methods, and few numerical simulation studies have been found in the literature. In experimental testing, the lap fillet welded joints with different load angles were often considered. In the high-temperature study, the factors of temperatures, rate of loading and cooling regimes were investigated in multiple research studies. Compared to ambient temperature, the strength, stiffness, and ductility of fillet welded joints were found to decrease with increasing temperatures. Although the failure modes observed are almost the same. However, the failure mechanism of fillet welded joints under fatigue loading at elevated temperatures is still unclear, and the knowledge of fatigue strength and behaviour will provide great value to the engineering structures safety. Therefore, the studies on fatigue performance at elevated temperatures required more attention.

## 7. Current Research Limitation and Future Direction

This paper summarises and reviews the major research from over 100 published papers on fillet welded joints. Based on the critical review of experimental and numerical studies reported in the literature, the current research limitations and future directions are discussed below:

- Very few numerical studies were done to investigate the effect of the gap between the loaded plates in fillet welded joints under static loading, however, not even a single experimental study was reported. Hence, more efforts could be put into better understanding the effect of the gap between the loaded plates on static and fatigue performance.
- Regarding the thermal performance, some experimental studies are available (with no numerical studies being reported in the literature) on fillet welded joints under static loading with limited influencing factors and there is no available research investigating the fatigue strength of such joints. Hence, further experimental and numerical studies are required to understand the strength and behaviour of fillet welded joints under static and fatigue loading at elevated temperatures, including the effect of weld geometry, weld type, loading direction, grade of steel, weld penetration depth, post-welded treatment, weld process, loading rates, cooling regimes, and temperatures.
- There is a lack of research on the static and fatigue strength of fillet welded joints at sub-zero temperatures. Additionally, their design strength has not been adequately investigated. Therefore, future research is required in this field.

## Figures and Tables

**Table 1:** Fillet welded joints under static loading.

Author Name	Year	Type of Steel	Type of Fillet Weld	Joint Type	Type of Investigation	FEA Model Type and Software
Butler and Kulak [17]	1971	CSA G40.12	Transverse, longitudinal and inclined	Lap joint	Experimental tests	-
Teh et al. [18]	2000	G450	Transverse and longitudinal	Lap joint	Experimental tests	-
Ng et al. [19]	2002	CSA G40.21	Transverse	Lap and cruciform joint	Experimental tests	-
Deng et al. [20]	2003	CSA G40.21	Transverse, longitudinal and inclined	Lap joint	Experimental tests	-
Callele et al. [21]	2005	CSA G40.21	Transverse, longitudinal and inclined	Lap joint	Experimental tests	-
Kuhlmann et al. [22]	2008	S235, S355J2, S460M and S690Q	Transverse and longitudinal	Lap and cruciform joint	Experimental tests and FEA	(ANSYS)
Khurshid et al. [5]	2012	S600 and S690	Transverse	Cruciform joint	Experimental tests and FEA	-
Bjork et al. [23]	2012	S960	Transverse and longitudinal	Lap and cruciform joint	Experimental tests and FEA	3D model (FEMAP)
Bjork et al. [1]	2014	S690Q	Transverse and longitudinal	Lap joint	Experimental tests	-
Lu et al. [3]	2015	Steel yield stress of 490MPa and 660 MPa	Transverse and longitudinal	Lap joint	Experimental tests and FEA	2D and 3D model (ABAQUS)
Sachin and Vyavahare [6]	2015	Steel yield stress of 250 MPa	Transverse	T-Joint	FEA	-
Barsoum and Khurshid [24]	2017	Steel yield stress of 350 to 960 MPa	Transverse	Cruciform joint	Experimental tests and FEA	2D model (ABAQUS)
Shi and Chen [25]	2017	Steel yield stress of 460 MPa	Transverse	Lap joint	Experimental tests and FEA	3D model (ABAQUS)
Fortan et al. [26]	2017	Austenitic (304L, 316L) and duplex steel	Transverse and longitudinal	Lap Joint	Experimental tests	-
Lee et al. [27]	2017	Austenitic steel (304L)	Transverse, longitudinal and full-length	Lap Joint	Experimental tests	-
Torabian et al. [4]	2018	Steel yield stress of 384.3 to 440.3 MPa	Transverse	Lap joint	Experimental tests	-
Collin and Johansson [28]	2018	Steel yield stress of 779 MPa and 789 MPa	Transverse and longitudinal	Lap joint	Experimental tests	-
Sun et al. [29]	2019	S690Q	Transverse	Lap and cruciform joint	Experimental tests	-
Yang et al. [30]	2019	Duplex steel	Transverse and longitudinal	Lap Joint	Experimental tests and FEA	3D model (ANSYS)
Lee et al. [31]	2019	Austenitic steel (304L)	Transverse and longitudinal	Lap Joint	Experimental tests and 13 FEA	3D model (ABAQUS)
Fortan et al. [32]	2020	Austenitic (304L, 316L), duplex steel	Transverse and longitudinal	Lap Joint	Experimental tests	-
Ran et al. [33]	2021	S690Q	Longitudinal	Lap joint	Experimental tests and FEA	3D model (ABAQUS)
Cho et.al [34]	2021	Duplex steel	Full-length	Lap Joint	Experimental tests and FEA	3D model (ABAQUS)
Ran et al. [35]	2022	S690Q	Transverse	Lap and cruciform joint	FEA	2D model (ABAQUS)

**Table 2:** Fatigue strength of fillet welded joints.

Author Name	Year	Type of Steel	Joint Type	Stress Ratio (R)	Frequency (HZ)	Type of Investigation	FEA Model Type and Software
Ohta et al. [61]	1994	SM50B	Cruciform joint	0	-	Experimental tests	-
Singh et al. [62]	2002	Stainless steel (304L)	Cruciform joint	0	30	Experimental tests	-
Infante et al. [63]	2003	Duplex steel (2205)	Cruciform joint	0.05,0.5	10–20	Experimental tests and FEA	2D model (ABAQUS)
Metrovich and Fisher [64]	2005	Super austenitic AL-6XN	I beam	-	-	Experimental tests	-
Kainuma and Kim [65]	2005	S490	Cruciform joint	-	4 to 20	Experimental tests and FEA	2D model
Kainuma and Mori [8]	2006	S400	Cruciform joint	-	4 to 20	Experimental tests and FEA	2D model
Caccese et al. [66]	2006	HSLA-65	Cruciform joint	-	-	Experimental tests and FEA	2D model (ANSYS)
Kainuma and Mori [67]	2008	S400	Cruciform joint	0	4 to 20	Experimental tests and FEA	2D model
Baik et al. [68]	2008	SM400 A	T-Joint and cruciform joint	-	-	Experimental tests and FEA	2D model (COSMOS)
Lee et al. [69]	2009	SM490 A	Cruciform	0	5	Experimental tests	-
Hanji et al. [7]	2013	S490 and S550	Cruciform joint	0.05	10	Experimental tests and FEA	2D model (ABAQUS)
Mohamed et al. [70]	2015	Steel yield stress of 550 MPa	Cruciform joint	0	-	FEA	2D and 3d models (ANSYS)
Corigliano et al. [71]	2015	S235JR	T-Joint	-	0.1 and 1	Experimental tests and FEA	3D model (ANSYS)
Vishnuvaradhan et al. [72]	2016	Steel yield stress of 306 and 468 MPa	Cruciform joint	-	-	Experimental tests and FEA	3D model (ABAQUS)
Ahola et al. [73]	2017	S960	T joint and cruciform joint	-	-	Experimental tests and FEA	2D model
Skriko et al. [74]	2017	S960	Cruciform joint	0.1, 0.25 to 0.60	2 to 5.2	Experimental tests and FEA	2D model (ABAQUS)
Mecseri and Kovesdi [75]	2017	S235 and S420	Cruciform joint	-	-	Experimental tests	-
Zong et al. [76]	2017	Q345qD	Cruciform joint	0.1	-	Experimental tests and FEA	2D and 3D model (ABAQUS)
Bjork et al. [77]	2018	Duplex steel (2205) and super duplex steel (2507)	Cruciform joint	-	-	Experimental tests	2D model (FEMAP)
Shiozaki et al. [9]	2018	Steel yield stress of 980 MPa	Lap joint	0	10 to 14	Experimental tests and FEA	2D model
Thomsen and Andreasen [78]	2018	S1100	T-Joint	−1	1 to 3	Experimental tests	-
Braun et al. [79]	2019	S235 and S500	Cruciform joint	0 to 0.15	33	Experimental tests	-
Karabulut et al. [80]	2020	Duplex steel	Cruciform joint	-	-	Experimental tests and FEA	3D model (ABAQUS)
Mettanen et al. [81]	2020	S960	Cruciform joint	0.1 to 0.6	-	Experimental tests and FEA	2D model (ANSYS)
Ahola et al. [82]	2020	S1100	T-Joint and cruciform joint	0.1 and 0.5	-	Experimental tests	-
Peng et al. [83]	2021	Austenitic (S30403)	Cruciform joint	0.5, 0.1	15	Experimental tests	
Karabulut et al. [84]	2021	Duplex steel	Cruciform joint	0.3	10	Experimental tests and FEA	3D model (ABAQUS)
Shin et al. [85]	2021	Steel yield stress of 490 MPa	Cruciform joint	0.1	5	Experimental tests and FEA	3D model
Chatzopoulou et al. [2]	2021	S275 and S355	L-joint	-	-	Experimental tests and FEA	3D model (ABAQUS)

**Table 3:** Thermal performance of fillet welded joints under static loading.

Author	Year	Type of Weld	Type of Investigation	Fire Condition	Temperature Range
Colon et al. [12]	2009	Transverse	Experimental tests	At elevated and post-elevated temperatures	20 to 870 °C
Zang et al. [16]	2017	Transverse and longitudinal	Experimental tests	At post-elevated temperatures	20 to 800 °C
Ghor and Hantouche [13]	2021	Transverse, longitudinal and inclined	Experimental tests	At post-elevated temperatures	20 to 700 °C
Ghor et al. [10]	2021	Transverse	Experimental tests	At elevated temperatures	20 to 700 °C
Chen and Chen [11]	2022	Transverse and longitudinal	Experimental tests	At elevated temperatures	20 to 1100 °C
Ghor and Hantouche [14]	2022	Transverse, longitudinal and inclined	Experimental tests	At post-elevated temperatures	20 to 900 °C

**Table 4:** Static strength of fillet welded joints on hollow sections.

Author Name	Year	Yield Stress of Base Metal	Type of Weld	Joint Type	Connection Type	Type of Investigation	FEA Type and Software
Zhao et al. [115]	1999	450 MPa	Longitudinal	Lap joint	RHS-to-rigid plate	Experimental tests and FEA	2D model (ABAQUS)
Ling et al. [114]	2002	1500 MPa	Longitudinal	Lap joint	CHS-to-rigid plate	Experiment tests	-
Jiao and Zhao [113]	2004	1500 MPa	Transverse	T-joint	CHS-to-rigid plate	Experiment tests	-
Packer et al. [111]	2016	350 MPa	Transverse	T-Joint	RHS-to-rigid plate and CHS-to-rigid plate	Experiment tests	-
Tousignant and Packer [108]	2017	373 to 431 MPa	Transverse	X-Joint	CHS-to-CHS	Experiment tests	-
Tousignant and Packer [109]	2017	431 to 517 MPa	Transverse	X-Joint	CHS-to-CHS	FEA	3D model (ANSYS)
Tousignant and Packer [110]	2019	385 to 431 MPa	Transverse	T-Joint and X-Joint	CHS-to-rigid plate and CHS-to-CHS	Experiment tests and FEA	3D model (ANSYS)
Xin et al. [112]	2021	700 MPa	Transverse	K-joint	RHS-to-RHS	FEA	3D model (ABAQUS)

**Table 5:** Fatigue strength of fillet welded joints on hollow sections.

Author Name	Year	Yield Stress of Base Metal	Joint Type	Connection Type	Stress Ratio	Frequency (HZ)	Type of Investigation
Mashiri et al. [116]	2002	350 to 450 MPa	T-Joint	SHS-to-rigid plate	0.5 and 0.1	1	Experimental tests
Mashiri et al. [117]	2002	350 to 450 MPa	T-Joint	SHS-to-SHS	0.1	-	Experimental tests
Tong et al. [121]	2006	350 to 430 MPa	T-Joint	CHS-to-SHS	0.1	1	Experimental tests and FEA
Mashiri and Zhao [119]	2007	350 MPa	T-Joint	CHS-to-rigid plate	0.1	-	Experimental tests
Jiao et al. [118]	2013	1500 MPa	T-Joint	CHS-to-rigid plate	0.1	-	Experimental tests

## Data Availability

The data presented in this paper has been properly cited.
